# Unbiased Feature Selection in Learning Random Forests for High-Dimensional Data

**DOI:** 10.1155/2015/471371

**Published:** 2015-03-24

**Authors:** Thanh-Tung Nguyen, Joshua Zhexue Huang, Thuy Thi Nguyen

**Affiliations:** ^1^Shenzhen Key Laboratory of High Performance Data Mining, Shenzhen Institutes of Advanced Technology, Chinese Academy of Sciences, Shenzhen 518055, China; ^2^University of Chinese Academy of Sciences, Beijing 100049, China; ^3^School of Computer Science and Engineering, Water Resources University, Hanoi 10000, Vietnam; ^4^College of Computer Science and Software Engineering, Shenzhen University, Shenzhen 518060, China; ^5^Faculty of Information Technology, Vietnam National University of Agriculture, Hanoi 10000, Vietnam

## Abstract

Random forests (RFs) have been widely used as a powerful classification method. However, with the randomization in both bagging samples and feature selection, the trees in the forest tend to select uninformative features for node splitting. This makes RFs have poor accuracy when working with high-dimensional data. Besides that, RFs have bias in the feature selection process where multivalued features are favored. Aiming at debiasing feature selection in RFs, we propose a new RF algorithm, called xRF, to select good features in learning RFs for high-dimensional data. We first remove the uninformative features using *p*-value assessment, and the subset of unbiased features is then selected based on some statistical measures. This feature subset is then partitioned into two subsets. A feature weighting sampling technique is used to sample features from these two subsets for building trees. This approach enables one to generate more accurate trees, while allowing one to reduce dimensionality and the amount of data needed for learning RFs. An extensive set of experiments has been conducted on 47 high-dimensional real-world datasets including image datasets. The experimental results have shown that RFs with the proposed approach outperformed the existing random forests in increasing the accuracy and the AUC measures.

## 1. Introduction

Random forests (RFs) [1] are a nonparametric method that builds an ensemble model of decision trees from random subsets of features and bagged samples of the training data.

RFs have shown excellent performance for both classification and regression problems. RF model works well even when predictive features contain irrelevant features (or noise); it can be used when the number of features is much larger than the number of samples. However, with randomizing mechanism in both bagging samples and feature selection, RFs could give poor accuracy when applied to high dimensional data. The main cause is that, in the process of growing a tree from the bagged sample data, the subspace of features randomly sampled from thousands of features to split a node of the tree is often dominated by uninformative features (or noise), and the tree grown from such bagged subspace of features will have a low accuracy in prediction which affects the final prediction of the RFs. Furthermore, Breiman et al. noted that feature selection is biased in the classification and regression tree (CART) model because it is based on an information criteria, called multivalue problem [2]. It tends in favor of features containing more values, even if these features have lower importance than other ones or have no relationship with the response feature (i.e., containing less missing values, many categorical or distinct numerical values) [3, 4].

In this paper, we propose a new random forests algorithm using an unbiased feature sampling method to build a good subspace of unbiased features for growing trees. We first use random forests to measure the importance of features and produce raw feature importance scores. Then, we apply a statistical Wilcoxon rank-sum test to separate informative features from the uninformative ones. This is done by neglecting all uninformative features by defining threshold *θ*; for instance, *θ* = 0.05. Second, we use the Chi-square statistic test (*χ* ^2^) to compute the related scores of each feature to the response feature. We then partition the set of the remaining informative features into two subsets, one containing highly informative features and the other one containing weak informative features. We independently sample features from the two subsets and merge them together to get a new subspace of features, which is used for splitting the data at nodes. Since the subspace always contains highly informative features which can guarantee a better split at a node, this feature sampling method enables avoiding selecting biased features and generates trees from bagged sample data with higher accuracy. This sampling method also is used for dimensionality reduction, the amount of data needed for training the random forests model. Our experimental results have shown that random forests with this weighting feature selection technique outperformed recently the proposed random forests in increasing of the prediction accuracy; we also applied the new approach on microarray and image data and achieved outstanding results.

The structure of this paper is organized as follows. In Section 2, we give a brief summary of related works. In Section 3 we give a brief summary of random forests and measurement of feature importance score.  Section 4 describes our new proposed algorithm using unbiased feature selection.  Section 5 provides the experimental results, evaluations, and comparisons.  Section 6 gives our conclusions.

## 2. Related Works

Random forests are an ensemble approach to make classification decisions by voting the results of individual decision trees. An ensemble learner with excellent generalization accuracy has two properties, high accuracy of each component learner and high diversity in component learners [5]. Unlike other ensemble methods such as bagging [1] and boosting [6, 7], which create basic classifiers from random samples of the training data, the random forest approach creates the basic classifiers from randomly selected subspaces of data [8, 9]. The randomly selected subspaces increase the diversity of basic classifiers learnt by a decision tree algorithm.

Feature importance is the importance measure of features in the feature selection process [1, 10–14]. In RF frameworks, the most commonly used score of importance of a given feature is the mean error of a tree in the forest when the observed values of this feature are randomly permuted in the* out-of-bag* samples. Feature selection is an important step to obtain good performance for an RF model, especially in dealing with high dimensional data problems.

For feature weighting techniques, recently Xu et al. [13] proposed an improved RF method which uses a novel feature weighting method for subspace selection and therefore enhances classification performance on high dimensional data. The weights of feature were calculated by information gain ratio or *χ* ^2^-test; Ye et al. [14] then used these weights to propose a stratified sampling method to select feature subspaces for RF in classification problems. Chen et al. [15] used a stratified idea to propose a new clustering method. However, implementation of the random forest model suggested by Ye et al. is based on a binary classification setting, and it uses linear discriminant analysis as the splitting criteria. This stratified RF model is not efficient on high dimensional datasets with multiple classes. With the same way for solving two-class problem, Amaratunga et al. [16] presented a feature weighting method for subspace sampling to deal with microarray data, the *t*-test of variance analysis is used to compute weights for the features. Genuer et al. [12] proposed a strategy involving a ranking of explanatory features using the RFs score weights of importance and a stepwise ascending feature introduction strategy. Deng and Runger [17] proposed a guided regularized RF (GRRF), in which weights of importance scores from an ordinary random forest (RF) are used to guide the feature selection process. They found that the regularized least subset selected by their GRRF with minimal regularization ensures better accuracy than the complete feature set. However, regular RF was used as a classifier due to the fact that regularized RF may have higher variance than RF because the trees are correlated.

Several methods have been proposed to correct bias of importance measures in the feature selection process in RFs to improve the prediction accuracy [18–21]. These methods intend to avoid selecting an uninformative feature for node splitting in decision trees. Although the methods of this kind were well investigated and can be used to address the high dimensional problem, there are still some unsolved problems, such as the need to specify in advance the probability distributions, as well as the fact that they struggle when applied to large high dimensional data.

In summary, in the reviewed approaches, the gain at higher levels of the tree is weighted differently than the gain at lower levels of the tree. In fact, at lower levels of the tree, the gain is reduced because of the effect of splits on different features at higher levels of the tree. That affects the final prediction performance of RFs model. To remedy this, in this paper we propose a new method for unbiased feature subsets selection in high dimensional space to build RFs. Our approach differs from previous approaches in the techniques used to partition a subset of features. All uninformative features (considered as noise) are removed from the system and the best feature set, which is highly related to the response feature, is found using a statistical method. The proposed sampling method always provides enough highly informative features for the subspace feature at any levels of the decision trees. For the case of growing an RF model on data without noise, we used* in-bag* measures. This is a different importance score of features, which requires less computational time compared to the measures used by others. Our experimental results showed that our approach outperformed recently the proposed RF methods.

## 3. Background

### 3.1. Random Forest Algorithm

Given a training dataset *ℒ* = {(*X*_*i*_, *Y*_*i*_)_*i*=1_^*N*^∣*X*_*i*_ ∈ *ℛ* ^*M*^, *Y* ∈ {1,2,…, *c*}}, where *X*_*i*_ are features (also called predictor variables), *Y* is a class response feature, *N* is the number of training samples, and *M* is the number of features and a random forest model RF described in Algorithm 1, let ${\hat{Y}}^{k}$ be the prediction of tree *T*_*k*_ given input *X*. The prediction of random forest with *K* trees is

(1) $$\begin{array}{c} \hat{Y}=\text{majority}\;\;\text{vote}\;\;{\left\{{\hat{Y}}^{k}\right\}}_{1}^{K}. \end{array}$$

Since each tree is grown from a bagged sample set, it is grown with only two-thirds of the samples in *ℒ*, called* in-bag* samples. About one-third of the samples is left out and these samples are called* out-of-bag* (OOB) samples which are used to estimate the prediction error.

The OOB predicted value is ${\hat{Y}}^{\text{OOB}}=(1/\left\|{𝒪}_{i^{\prime }}\right\|)\sum_{k\in {𝒪}_{i^{\prime }}}{\hat{Y}}^{k}$ where *𝒪*_*i*′_ = *ℒ*∖*𝒪*_*i*_, *i* and *i*′ are in-bag and out-of-bag sampled indices, ‖*𝒪*_*i*′_‖ is the size of OOB subdataset, and the OOB prediction error is

(2) $$\begin{array}{c} {\hat{\text{Err}}}^{\text{OOB}}=\frac{1}{N_{\text{OOB}}}\overset{N_{\text{OOB}}}{\underset{i=1}{\sum }}E\left(Y,{\hat{Y}}^{\text{OOB}}\right), \end{array}$$

where *ℰ*(·) is an error function and *N*_OOB_ is OOB samples' size.

### 3.2. Measurement of Feature Importance Score from an RF

Breiman presented a permutation technique to measure the importance of features in the prediction [1], called an* out-of-bag* importance score. The basic idea for measuring this kind of importance score of features is to compute the difference between the original mean error and the randomly permuted mean error in OOB samples. The method rearranges stochastically all values of the *j*th feature in OOB for each tree and uses the RF model to predict this permuted feature and get the mean error. The aim of this permutation is to eliminate the existing association between the *j*th feature and *Y* values and then to test the effect of this on the RF model. A feature is considered to be in a strong association if the mean error decreases dramatically.

The other kind of feature importance measure can be obtained when the random forest is growing. This is described as follows. At each node *t* in a decision tree, the split is determined by the decrease in node impurity Δ*R*(*t*). The node impurity *R*(*t*) is the gini index. If a subdataset in node *t* contains samples from *c* classes, gini(*t*) is defined as

(3) $$\begin{array}{c} R\left(t\right)=1-\overset{c}{\underset{j=1}{\sum }}{\hat{p}}_{j}^{2}, \end{array}$$

where ${\hat{p}}_{j}^{2}$ is the relative frequency of class *j* in *t*. Gini(*t*) is minimized if the classes in *t* are skewed. After splitting *t* into two child nodes *t*_1_ and *t*_2_ with sample sizes *N*_1_(*t*) and *N*_2_(*t*), the gini index of the split data is defined as

(4) $$\begin{array}{c} \text{Gin}{\text{i}}_{\text{split}}\left(t\right)=\frac{N_{1}\left(t\right)}{N\left(t\right)}\text{Gini}\left(t_{1}\right)+\frac{N_{2}\left(t\right)}{N\left(t\right)}\text{Gini}\left(t_{2}\right). \end{array}$$

The feature providing smallest Gini_split_(*t*) is chosen to split the node. The importance score of feature *X*_*j*_ in a single decision tree *T*_*k*_ is

(5) $$\begin{array}{c} \text{I}{\text{S}}_{k}\left(X_{j}\right)=\underset{t\in T_{k}}{\sum }\Delta R\left(t\right), \end{array}$$

and it is computed over all *K* trees in a random forest, defined as

(6) $$\begin{array}{c} \text{IS}\left(X_{j}\right)=\frac{1}{K}\overset{K}{\underset{k=1}{\sum }}\text{I}{\text{S}}_{k}\left(X_{j}\right). \end{array}$$

It is worth noting that a random forest uses* in-bag* samples to produce a kind of importance measure, called an* in-bag* importance score. This is the main difference between the* in-bag* importance score and an* out-of-bag* measure, which is produced with the decrease of the prediction error using RF in OOB samples. In other words, the* in-bag* importance score requires less computation time than the* out-of-bag* measure.

## 4. Our Approach

### 4.1. Issues in Feature Selection on High Dimensional Data

When Breiman et al. suggested the classification and regression tree (CART) model, they noted that feature selection is biased because it is based on an information gain criteria, called multivalue problem [2]. Random forest methods are based on CART trees [1]; hence this bias is carried to random forest RF model. In particular, the importance scores can be biased when very high dimensional data contains multiple data types. Several methods have been proposed to correct bias of feature importance measures [18–21]. The conditional inference framework (referred to as cRF [22]) could be successfully applied for both the null and power cases [19, 20, 22]. The typical characteristic of the power case is that only one predictor feature is important, while the rest of the features are redundant with different cardinality. In contrast, in the null case all features used for prediction are redundant with different cardinality. Although the methods of this kind were well investigated and can be used to address the multivalue problem, there are still some unsolved problems, such as the need to specify in advance the probability distributions, as well as the fact that they struggle when applied to high dimensional data.

Another issue is that, in high dimensional data, when the number of features is large, the fraction of importance features remains so small. In this case the original RF model which uses simple random sampling is likely to perform poorly with small *m*, and the trees are likely to select an uninformative feature as a split too frequently (*m* denotes a subspace size of features). At each node *t* of a tree, the probability of uninformative feature selection is too high.

To illustrate this issue, let *G* be the number of noisy features, denote by *M* the total number of predictor features, and let the features *M* − *G* be important ones which have a high correlation with *Y* values. Then, if we use simple random sampling when growing trees to select a subset of *m* features (*m* ≪ *M*), the total number of possible uninformative a *𝒞*_*M*−*G*_^*m*^ and the total number of all subset features is *𝒞*_*M*_^*m*^. The probability distribution of selecting a subset of *m* (*m* > 1) important features is given by

(7) CM−GmCMm=M−GM−G−1⋯M−G−m+1MM−1⋯M−m+1=1−G/M⋯1−G/M−m/M+1/M1−1/M⋯1−m/M+1/M≃1−GMm.

Because the fraction of important features is too small, the probability in (7) tends to 0, which means that the important features are rarely selected by the simple sampling method in RF [1]. For example, with 5 informative and 5000 noise or uninformative features, assuming $m=\sqrt{\left(5+5000\right)}≃70$, the probability of an informative feature to be selected at any split is 0.068.

### 4.2. Bias Correction for Feature Selection and Feature Weighting

The bias correction in feature selection is intended to make the RF model to avoid selecting an uninformative feature. To correct this kind of bias in the feature selection stage, we generate shadow features to add to the original dataset. The shadow features set contains the same values, possible cut-points, and distribution with the original features but have no association with *Y* values. To create each shadow feature, we rearrange the values of the feature in the original dataset *R* times to create the corresponding shadow. This disturbance of features eliminates the correlations of the features with the response value but keeps its attributes. The shadow feature participates only in the competition for the best split and makes a decrease in the probability of selecting this kind of uninformative feature. For the feature weight computation, we first need to distinguish the important features from the less important ones. To do so, we run a defined number of random forests to obtain raw importance scores, each of which is obtained using (6). Then, we use Wilcoxon rank-sum test [23] that compares the importance score of a feature with the maximum importance scores of generated noisy features called shadows. The shadow features are added to the original dataset and they do not have prediction power to the response feature. Therefore, any feature whose importance score is smaller than the maximum importance score of noisy features is considered less important. Otherwise, it is considered important. Having computed the Wilcoxon rank-sum test, we can compute the *p*-value for the feature. The *p*-value of a feature in Wilcoxon rank-sum test is assigned a weight with a feature *X*_*j*_, *p*-value ∈  [0,1], and this weight indicates the importance of the feature in the prediction. The smaller the *p*-value of a feature, the more correlated the predictor feature to the response feature, and therefore the more powerful the feature in prediction. The feature weight computation is described as follows.

Let *M* be the number of features in the original dataset, and denote the feature set as *𝒮*_*X*_ = {*X*_*j*_, *j* = 1,2,…, *M*}. In each replicate *r*  (*r* = 1,2,…, *R*), shadow features are generated from features *X*_*j*_ in *𝒮*_**X**_, and we randomly permute all values of *X*_*j*_ *R* times to get a corresponding shadow feature *A*_*j*_; denote the shadow feature set as *𝒮*_*A*_ = {*A*_*j*_}_1_^*M*^. The extended feature set is denoted by *𝒮*_*X*,*A*_ = {*𝒮*_*X*_, *𝒮*_*A*_}.

Let the importance score of *𝒮*_*X*,*A*_ at replicate *r* be IS_*X*,*A*_^*r*^ = {IS_*X*_^*r*^, IS_*A*_^*r*^} where IS_*X*_*j*__^*r*^ and IS_*A*_*j*__^*r*^ are the importance scores of *X*_*j*_ and *A*_*j*_ at the *r*th replicate, respectively. We built a random forest model RF from the *𝒮*_*X*,*A*_ dataset to compute 2*M* importance scores for 2*M* features. We repeated the same process *R* times to compute *R* replicates getting IS_*X*_*j*__ = {IS_*X*_*j*__^*r*^}_1_^*R*^ and IS_*A*_*j*__ = {IS_*A*_*j*__^*r*^}_1_^*R*^. From the replicates of shadow features, we extracted the maximum value from *r*th row of IS_*A*_*j*__ and put it into the comparison sample denoted by IS_*A*_^max⁡^. For each data feature *X*_*j*_, we computed Wilcoxon test and performed hypothesis test on ${\bar{\text{IS}}}_{X_{j}}>{\bar{\text{IS}}}_{A}^{\max}$ to calculate the *p*-value for the feature. Given a statistical significance level, we can identify important features from less important ones. This test confirms that if a feature is important, it consistently scores higher than the shadow over multiple permutations. This method has been presented in [24, 25].

In each node of trees, each shadow *A*_*j*_ shares approximately the same properties of the corresponding *X*_*j*_, but it is independent on *Y* and consequently has approximately the same probability of being selected as a splitting candidate. This feature permutation method can reduce bias due to different measurement levels of *X*_*j*_ according to *p*-value and can yield correct ranking of features according to their importance.

### 4.3. Unbiased Feature Weighting for Subspace Selection

Given all *p*-values for all features, we first set a significance level as the threshold *θ*, for instance *θ* = 0.05. Any feature whose *p*-value is greater than *θ* is considered a uninformative feature and is removed from the system; otherwise, the relationship with *Y* is assessed. We now consider the set of features $\tilde{X}$ obtained from *ℒ* after neglecting all uninformative features.

Second, we find the best subset of features which is highly related to the response feature; a measure correlation function $\chi^{2}(\tilde{X},Y)$ is used to test the association between the categorical response feature and each feature *X*_*j*_. Each observation is allocated to one cell of a two-dimensional array of cells (called a contingency table) according to the values of $\left(\tilde{X},Y\right)$. If there are *r* rows and *c* columns in the table and *N* is the number of total samples, the value of the test statistic is

(8) $$\begin{array}{c} \chi^{2}=\overset{r}{\underset{i=1}{\sum }}\;\overset{c}{\underset{j=1}{\sum }}\frac{{\left(O_{i,j}-E_{i,j}\right)}^{2}}{E_{i,j}}. \end{array}$$

For the test of independence, a chi-squared probability of less than or equal to 0.05 is commonly interpreted for rejecting the hypothesis that the row variable is independent of the column feature.

Let **X**_*s*_ be the best subset of features, we collect all feature *X*_*j*_ whose *p*-value is smaller or equal to 0.05 as a result from the *χ* ^2^ statistical test according to (8). The remaining features $\left\{\tilde{X}\setminus X_{s}\right\}$ are added to **X**_*w*_, and this approach is described in Algorithm 2. We independently sample features from the two subsets and put them together as the subspace features for splitting the data at any node, recursively. The two subsets partition the set of informative features in data without irrelevant features. Given **X**_*s*_ and **X**_*w*_, at each node, we randomly select *mtry* (*mtry* > 1) features from each group of features. For a given subspace size, we can choose proportions between highly informative features and weak informative features that depend on the size of the two groups. That is $mtry_{s}=\left\lceil mtry\times (\left\|X_{s}\right\|/\left\|\tilde{X}\right\|)\right\rceil$ and $mtry_{w}=\left\lfloor mtry\times (\left\|X_{w}\right\|/\left\|\tilde{X}\right\|)\right\rfloor$, where ‖**X**_*s*_‖ and ‖**X**_*w*_‖ are the number of features in the groups of highly informative features **X**_*s*_ and weak informative features **X**_*w*_, respectively. $\left\|\tilde{X}\right\|$ is the number of informative features in the input dataset. These are merged to form the feature subspace for splitting the node.

### 4.4. Our Proposed RF Algorithm

In this section, we present our new random forest algorithm called xRF, which uses the new unbiased feature sampling method to generate splits at the nodes of CART trees [2]. The proposed algorithm includes the following main steps: (i) weighting the features using the feature permutation method, (ii) identifying all unbiased features and partitioning them into two groups **X**_*s*_ and **X**_*w*_, (iii) building RF using the subspaces containing features which are taken randomly and separately from **X**_*s*_, **X**_*w*_, and (iv) classifying a new data. The new algorithm is summarized as follows.

1. Generate the extended dataset *𝒮*_**X**,*A*_ of 2*M* dimensions by permuting the corresponding predictor feature values for shadow features.
2. Build a random forest model RF from {*𝒮*_**X**,*A*_, *Y*} and compute *R* replicates of raw importance scores of all predictor features and shadows with RF. Extract the maximum importance score of each replicate to form the comparison sample IS_*A*_^max⁡^ of *R* elements.
3. For each predictor feature, take *R* importance scores and compute Wilcoxon test to get *p*-value, that is, the weight of each feature.
4. Given a significance level threshold *θ*, neglect all uninformative features.
5. Partition the remaining features into two subsets **X**_*s*_ and **X**_*w*_ described in Algorithm 2.
6. Sample the training set *ℒ* with replacement to generate bagged samples *ℒ*_1_, *ℒ*_2_,…, *ℒ*_*K*_.
7. For each *L*_*k*_, grow a CART tree *T*_*k*_ as follows.
  - At each node, select a subspace of *mtry* (*mtry* > 1) features randomly and separately from **X**_*s*_ and **X**_*w*_ and use the subspace features as candidates for splitting the node.
  - Each tree is grown nondeterministically, without pruning until the minimum node size *n*_min⁡_ is reached.
8. Given a *X* = *x*_new_, use (1) to predict the response value.

## 5. Experiments

### 5.1. Datasets

Real-world datasets including image datasets and microarray datasets were used in our experiments. Image classification and object recognition are important problems in computer vision. We conducted experiments on four benchmark image datasets, including the* Caltech* categories (http://www.vision.caltech.edu/html-files/archive.html) dataset, the* Horse* (http://pascal.inrialpes.fr/data/horses/) dataset, the extended* YaleB* database [26], and the* AT&T ORL* dataset [27].

For the* Caltech* dataset, we use a subset of 100 images from the* Caltech* face dataset and 100 images from the* Caltech* background dataset following the setting in ICCV (http://people.csail.mit.edu/torralba/shortCourseRLOC/). The extended* YaleB* database consists of 2414 face images of 38 individuals captured under various lighting conditions. Each image has been cropped to a size of 192 × 168 pixels and normalized. The* Horse* dataset consists of 170 images containing horses for the positive class and 170 images of the background for the negative class. The* AT&T ORL* dataset includes of 400 face images of 40 persons.

In the experiments, we use a bag of words for image features representation for the* Caltech* and the* Horse* datasets. To obtain feature vectors using bag-of-words method, image patches (subwindows) are sampled from the training images at the detected interest points or on a dense grid. A visual descriptor is then applied to these patches to extract the local visual features. A clustering technique is then used to cluster these, and the cluster centers are used as visual code words to form visual codebook. An image is then represented as a histogram of these visual words. A classifier is then learned from this feature set for classification.

In our experiments, traditional *k*-means quantization is used to produce the visual codebook. The number of cluster centers can be adjusted to produce the different vocabularies, that is, dimensions of the feature vectors. For the* Caltech* and Horse datasets, nine codebook sizes were used in the experiments to create 18 datasets as follows: {*CaltechM300*,* CaltechM500*,* CaltechM1000*,* CaltechM3000*,* CaltechM5000*,* CaltechM7000*,* CaltechM1000*,* CaltechM12000*,* CaltechM15000*}, and {*HorseM300*,* HorseM500*,* HorseM1000*,* HorseM3000*,* HorseM5000*,* HorseM7000*,* HorseM1000*,* HorseM12000*,* HorseM15000*}, where* M* denotes the number of codebook sizes.

For the face datasets, we use two type of features: eigenface [28] and the random features (randomly sample pixels from the images). We used four groups of datasets with four different numbers of dimensions {*M*30,  *M*56,  *M*120,  and *M*504}. Totally, we created 16 subdatasets as {*YaleB.EigenfaceM30*,* YaleB.EigenfaceM56*,* YaleB.EigenfaceM120*,* YaleB.EigenfaceM504*}, {*YaleB.RandomfaceM30*,* YaleB.RandomfaceM56*,* YaleB.RandomfaceM120*,* YaleB.RandomfaceM504*}, {*ORL.EigenfaceM30*,* ORL.EigenM56*,* ORL.EigenM120*,* ORL.EigenM504*}, and {*ORL.RandomfaceM30*,* ORL.RandomM56*,* ORL.RandomM120*,* ORL.RandomM504*}.

The properties of the remaining datasets are summarized in Table 1. The Fbis dataset was compiled from the archive of the Foreign Broadcast Information Service and the* La1s*,* La2s* datasets were taken from the archive of the Los Angeles Times for TREC-5 (http://trec.nist.gov/). The ten gene datasets are used and described in [11, 17]; they are always high dimensional and fall within a category of classification problems which deal with large number of features and small samples. Regarding the characteristics of the datasets given in Table 1, the proportion of the subdatasets, namely,* Fbis*,* La1s*,* La2s*, was used individually for a training and testing dataset.

### 5.2. Evaluation Methods

We calculated some measures such as error bound (*c*/*s*2), strength (*s*), and correlation ($\bar{\rho }$) according to the formulas given in Breiman's method [1]. The correlation measures indicate the independence of trees in a forest, whereas the average strength corresponds to the accuracy of individual trees. Lower correlation and higher strength result in a reduction of general error bound measured by (*c*/*s*2) which indicates a high accuracy RF model.

The two measures are also used to evaluate the accuracy of prediction on the test datasets: one is the area under the curve (AUC) and the other one is the test accuracy (Acc), defined as

(9) $$\begin{array}{c} \text{Acc}=\frac{1}{N}\overset{N}{\underset{i=1}{\sum }}I\left(Q\left(d_{i},y_{i}\right)-\underset{j\neq y_{i}}{\max}\;Q\left(d_{i},j\right)>0\right), \end{array}$$

where *I*(·) is the indicator function and *Q*(*d*_*i*_, *j*) = ∑_*k*=1_^*K*^ *I*(*h*_*k*_(*d*_*i*_) = *j*) is the number of votes for *d*_*i*_ ∈ *𝒟*_*t*_ on class *j*, *h*_*k*_ is the *k*th tree classifier, *N* is the number of samples in test data *𝒟*_*t*_, and *y*_*i*_ indicates the true class of *d*_*i*_.

### 5.3. Experimental Settings

The latest *R*-packages random Forest and RRF [29, 30] were used in *R* environment to conduct these experiments. The GRRF model was available in the RRF *R*-package. The wsRF model, which used weighted sampling method [13] was intended to solve classification problems. For the image datasets, the 10-fold cross-validation was used to evaluate the prediction performance of the models. From each fold, we built the models with 500 trees and the feature partition for subspace selection in Algorithm 2 was recalculated on each training fold dataset. The *mtry* and *n*_min⁡_ parameters were set to $\sqrt{M}$ and 1, respectively. The experimental results were evaluated in two measures AUC and the test accuracy according to (9).

We compared across a wide range the performances of the 10 gene datasets, used in [11]. The results from the application of GRRF, varSelRF, and LASSO logistic regression on the ten gene datasets are presented in [17]. These three gene selection methods used RF *R*-package [30] as the classifier. For the comparison of the methods, we used the same settings which are presented in [17], for the coefficient *γ* we used value of 0.1, because GR-RF(0.1) has shown competitive accuracy [17] when applied to the 10 gene datasets. The 100 models were generated with different seeds from each training dataset and each model contained 1000 trees. The *mtry* and *n*_min⁡_ parameters were of the same settings on the image dataset. From each of the datasets two-thirds of the data were randomly selected for training. The other one-third of the dataset was used to validate the models. For comparison, Breiman's RF method, the weighted sampling random forest wsRF model, and the xRF model were used in the experiments. The guided regularized random forest GRRF [17] and the two well-known feature selection methods using RF as a classifier, namely,* varSelRF* [31] and* LASSO logistic regression* [32], are also used to evaluate the accuracy of prediction on high-dimensional datasets.

In the remaining datasets, the prediction performances of the ten random forest models were evaluated, each one was built with 500 trees. The number of features candidates to split a node was *mtry* = ⌈log⁡_2_(*M*) + 1⌉. The minimal node size *n*_min⁡_ was 1. The xRF model with the new unbiased feature sampling method is a new implementation. We implemented the xRF model as multithread processes, while other models were run as single-thread processes. We used *R* to call the corresponding C/C++ functions. All experiments were conducted on the six 64-bit Linux machines, with each one being equipped with Intel *R* Xeon *R* CPU E5620 2.40 GHz, 16 cores, 4 MB cache, and 32 GB main memory.

### 5.4. Results on Image Datasets

**Figure 1:**
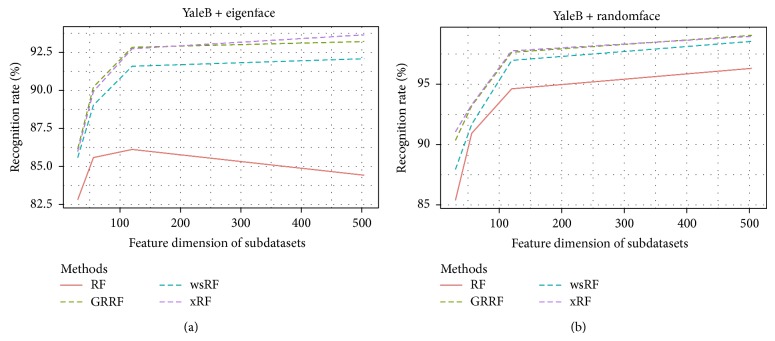
Recognition rates of the models on the YaleB subdatasets, namely, YaleB.EigenfaceM30, YaleB.EigenfaceM56, YaleB.EigenfaceM120, YaleB.EigenfaceM504, and YaleB.RandomfaceM30, YaleB.RandomfaceM56, YaleB.RandomfaceM120, and YaleB.RandomfaceM504.

**Figure 2:**
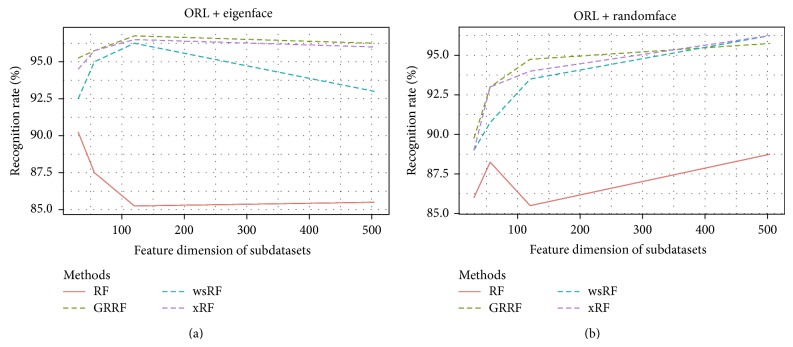
Recognition rates of the models on the ORL subdatasets, namely, ORL.EigenfaceM30, ORL.EigenM56, ORL.EigenM120, ORL.EigenM504, and ORL.RandomfaceM30, ORL.RandomM56, ORL.RandomM120, and ORL.RandomM504.

Figures 1 and 2 show the average accuracy plots of recognition rates of the models on different subdatasets of the datasets* YaleB* and* ORL*. The GRRF model produced slightly better results on the subdataset* ORL.RandomM120* and* ORL* dataset using eigenface and showed competitive accuracy performance with the xRF model on some cases in both* YaleB* and* ORL* datasets, for example,* YaleB.EigenM120*,* ORL.RandomM56*, and* ORL.RandomM120*. The reason could be that truly informative features in this kind of datasets were many. Therefore, when the informative feature set was large, the chance of selecting informative features in the subspace increased, which in turn increased the average recognition rates of the GRRF model. However, the xRF model produced the best results in the remaining cases. The effect of the new approach for feature subspace selection is clearly demonstrated in these results, although these datasets are not high dimensional.

**Figure 3:**
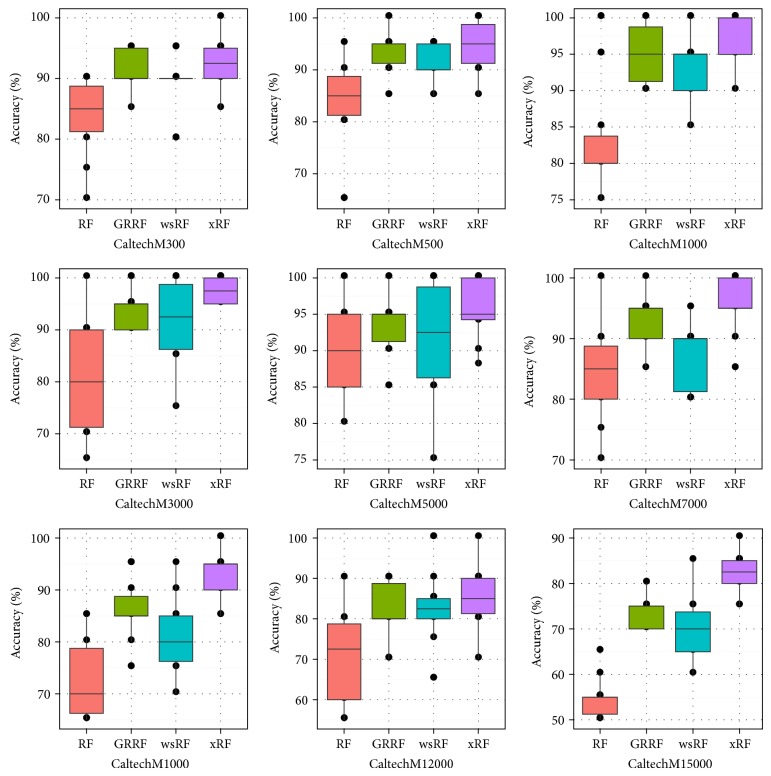
Box plots: the test accuracy of the nine Caltech subdatasets.

**Figure 4:**
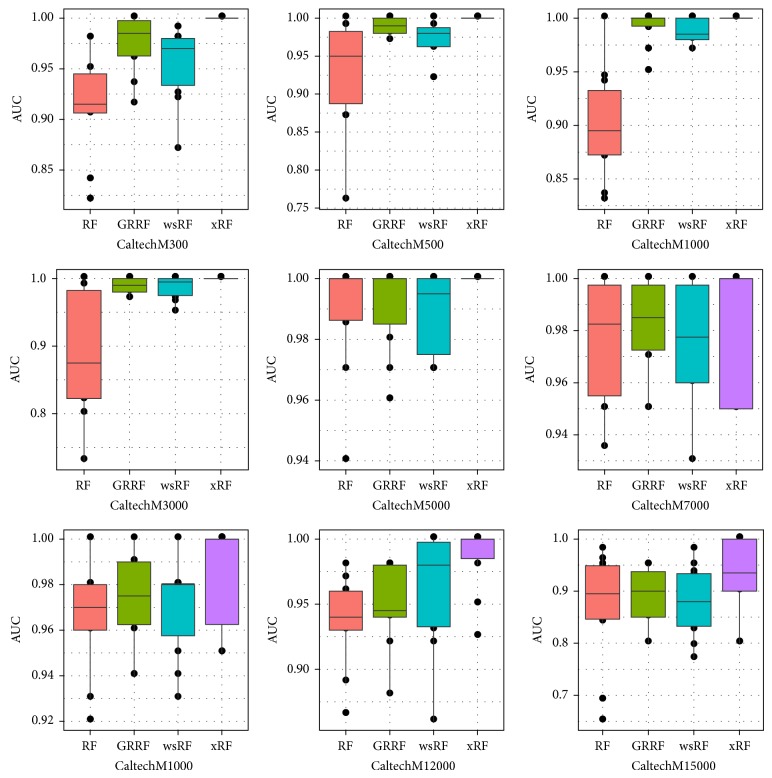
Box plots of the AUC measures of the nine Caltech subdatasets.

**Figure 5:**
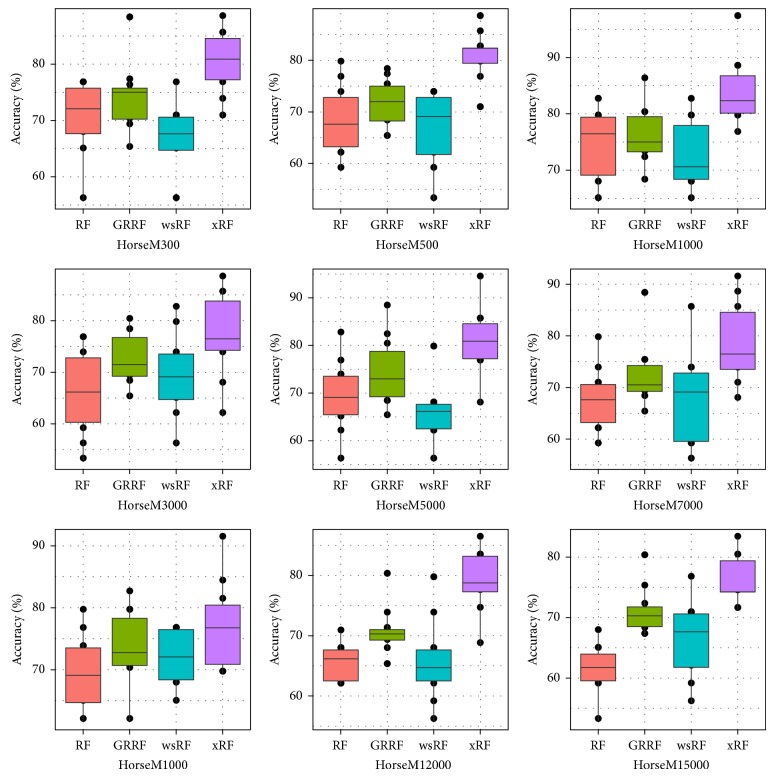
Box plots of the test accuracy of the nine Horse subdatasets.

**Figure 6:**
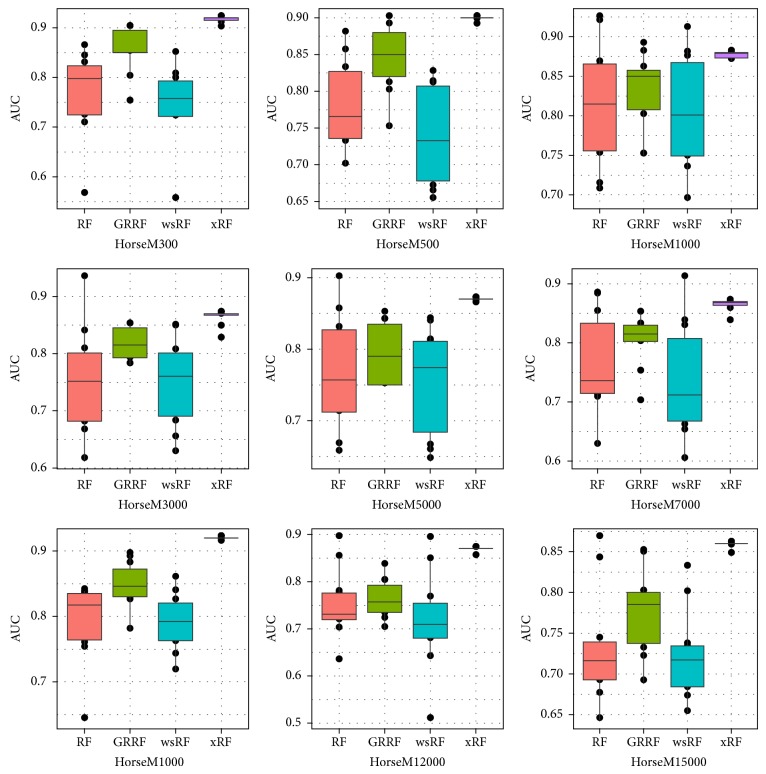
Box plots of the AUC measures of the nine Horse subdatasets.

Figures 3 and 5 present the box plots of the test accuracy (mean ± std-dev%); Figures 4 and 6 show the box plots of the AUC measures of the models on the 18 image subdatasets of the* Caltech* and* Horse*, respectively. From these figures, we can observe that the accuracy and the AUC measures of the models GRRF, wsRF, and xRF were increased on all high-dimensional subdatasets when the selected subspace *mtry* was not so large. This implies that when the number of features in the subspace is small, the proportion of the informative features in the feature subspace is comparatively large in the three models. There will be a high chance that highly informative features are selected in the trees so the overall performance of individual trees is increased. In Brieman's method, many randomly selected subspaces may not contain informative features, which affect the performance of trees grown from these subspaces. It can be seen that the xRF model outperformed other random forests models on these subdatasets in increasing the test accuracy and the AUC measures. This was because the new unbiased feature sampling was used in generating trees in the xRF model; the feature subspace provided enough highly informative features at any levels of the decision trees. The effect of the unbiased feature selection method is clearly demonstrated in these results.

Table 2 shows the results of *c*/*s*2 against the number of codebook sizes on the* Caltech* and* Horse* datasets. In a random forest, the tree was grown from a bagging training data. Out-of-bag estimates were used to evaluate the strength, correlation, and *c*/*s*2. The GRRF model was not considered in this experiment because this method aims to find a small subset of features, and the same RF model in *R*-package [30] is used as a classifier. We compared the xRF model with two kinds of random forest models RF and wsRF. From this table, we can observe that the lowest *c*/*s*2 values occurred when the wsRF model was applied to the* Caltech* dataset. However, the xRF model produced the lowest error bound on the *Horse* dataset. These results demonstrate the reason that the new unbiased feature sampling method can reduce the upper bound of the generalization error in random forests.

Table 3 presents the prediction accuracies (mean ± std-dev%) of the models on subdatasets* CaltechM3000*,* HorseM3000*,* YaleB.EigenfaceM504*,* YaleB.randomfaceM504*,* ORL.EigenfaceM504*, and* ORL.randomfaceM504*. In these experiments, we used the four models to generate random forests with different sizes from 20 trees to 200 trees. For the same size, we used each model to generate 10 random forests for the 10-fold cross-validation and computed the average accuracy of the 10 results. The GRRF model showed slightly better results on* YaleB.EigenfaceM504* with different tree sizes. The wsRF model produced the best prediction performance on some cases when applied to small subdatasets* YaleB.EigenfaceM504*,* ORL.EigenfaceM504*, and* ORL.randomfaceM504*. However, the xRF model produced, respectively, the highest test accuracy on the remaining subdatasets and AUC measures on high-dimensional subdatasets* CaltechM3000* and* HorseM3000*, as shown in Tables 3 and 4. We can clearly see that the xRF model also outperformed other random forests models in classification accuracy on most cases in all image datasets. Another observation is that the new method is more stable in classification performance because the mean and variance of the test accuracy measures were minor changed when varying the number of trees.

### 5.5. Results on Microarray Datasets

Table 5 shows the average test results in terms of accuracy of the 100 random forest models computed according to (9) on the gene datasets. The average number of genes selected by the xRF model, from 100 repetitions for each dataset, is shown on the right of Table 5, divided into two groups **X**_*s*_ (strong) and **X**_*w*_ (weak). These genes are used by the unbiased feature sampling method for growing trees in the xRF model. LASSO logistic regression, which uses the RF model as a classifier, showed fairly good accuracy on the two gene datasets* srbct* and* leukemia*. The GRRF model produced slightly better result on the* prostate* gene dataset. However, the xRF model produced the best accuracy on most cases of the remaining gene datasets.

**Figure 7:**
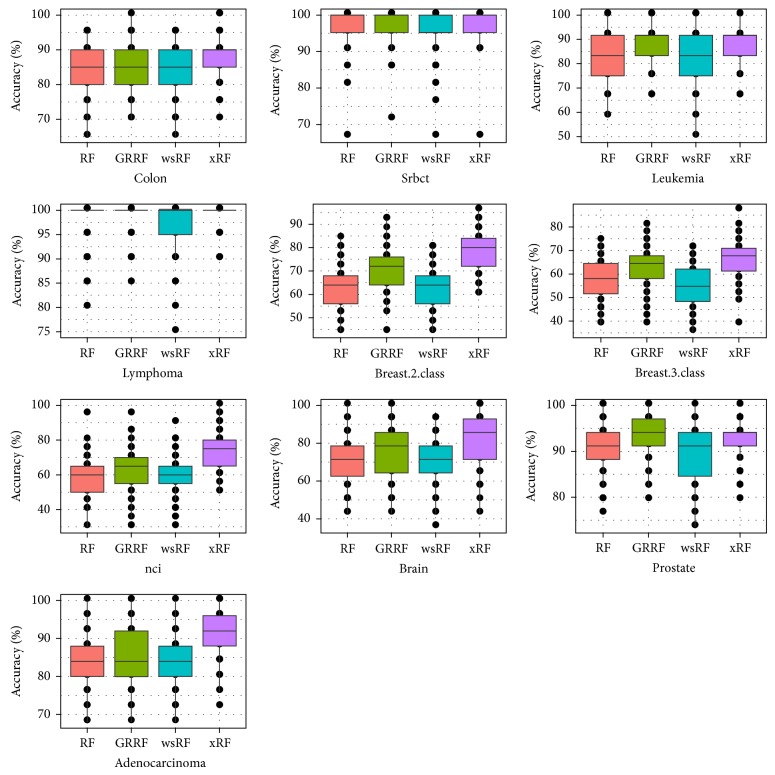
Box plots of test accuracy of the models on the ten gene datasets.

The detailed results containing the median and the variance values are presented in Figure 7 with box plots. Only the GRRF model was used for this comparison; the LASSO logistic regression and varSelRF method for feature selection were not considered in this experiment because their accuracies are lower than that of the GRRF model, as shown in [17]. We can see that the xRF model achieved the highest average accuracy of prediction on nine datasets out of ten. Its result was significantly different on the* prostate* gene dataset and the variance was also smaller than those of the other models.

**Figure 8:**
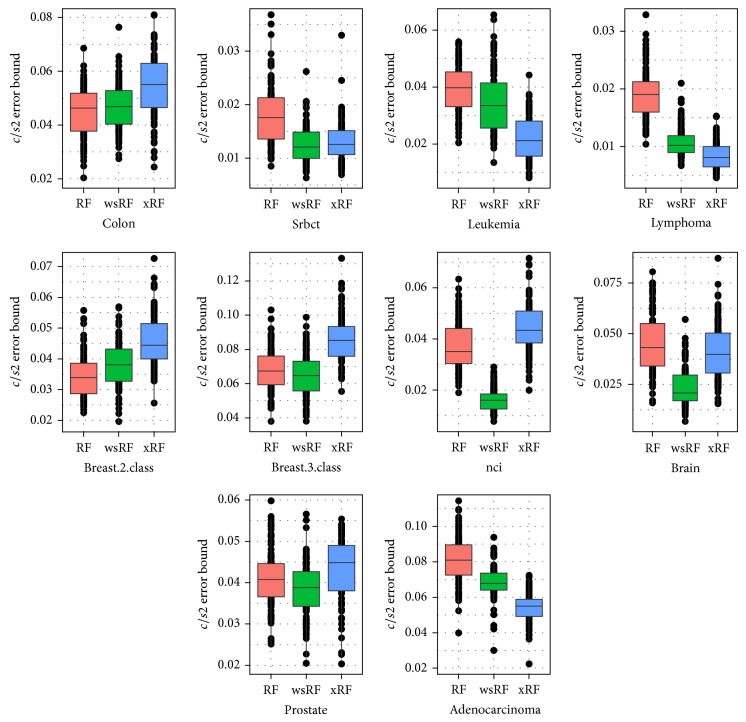
Box plots of (*c*/*s*2) error bound for the models applied to the 10 gene datasets.

Figure 8 shows the box plots of the (*c*/*s*2) error bound of the RF, wsRF, and xRF models on the ten gene datasets from 100 repetitions. The wsRF model obtained lower error bound rate on five gene datasets out of 10. The xRF model produced a significantly different error bound rate on two gene datasets and obtained the lowest error rate on three datasets. This implies that when the optimal parameters such as $mtry=\left\lceil \sqrt{M}\right\rceil$ and *n*_min⁡_ = 1 were used in growing trees, the number of genes in the subspace was not small and out-of-bag data was used in prediction, and the results were comparatively favored to the xRF model.

### 5.6. Comparison of Prediction Performance for Various Numbers of Features and Trees

Table 6 shows the average *c*/*s*2 error bound and accuracy test results of 10 repetitions of random forest models on the three large datasets. The xRF model produced the lowest error *c*/*s*2 on the dataset* La1s*, while the wsRF model showed the lower error bound on other two datasets* Fbis* and* La2s*. The RF model demonstrated the worst accuracy of prediction compared to the other models; this model also produced a large *c*/*s*2 error when the small subspace size *mtry* = ⌈log⁡_2_(*M*) + 1⌉ was used to build trees on the* La1s* and* La2s* datasets. The number of features in the **X**_*s*_ and **X**_*w*_ columns on the right of Table 6 was used in the xRF model. We can see that the xRF model achieved the highest accuracy of prediction on all three large datasets.

**Figure 9:**
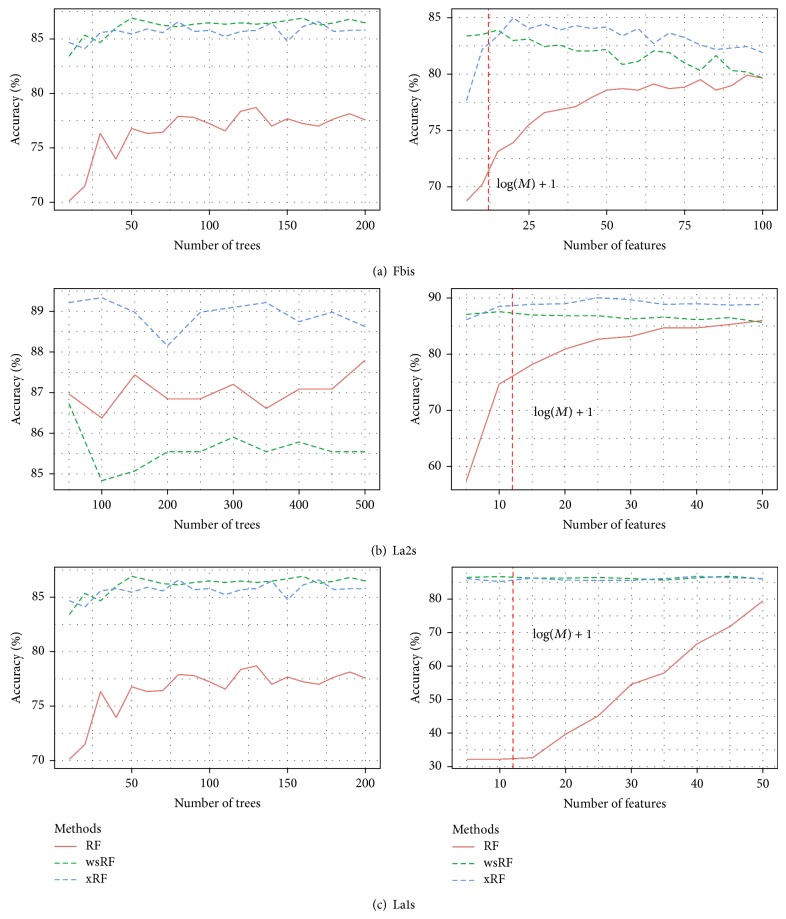
The accuracy of prediction of the three random forests models against the number of trees and features on the three datasets.

Figure 9 shows the plots of the performance curves of the RF models when the number of trees and features increases. The number of trees was increased stepwise by 20 trees from 20 to 200 when the models were applied to the* La1s* dataset. For the remaining data sets, the number of trees increased stepwise by 50 trees from 50 to 500. The number of random features in a subspace was set to $mtry=\left\lceil \sqrt{M}\right\rceil$. The number of features, each consisting of a random sum of five inputs, varied from 5 to 100, and for each, 200 trees were combined. The vertical line in each plot indicates the size of a subspace of features *mtry* = ⌈log⁡_2_(*M*) + 1⌉. This subspace was suggested by Breiman [1] for the case of low-dimensional datasets. Three feature selection methods, namely, GRRF, varSelRF, and LASSO, were not considered in this experiment. The main reason is that, when the *mtry* value is large, the computational time of the GRRF and varSelRF models required to deal with large high datasets was too long [17].

It can be seen that the xRF and wsRF models always provided good results and achieved higher prediction accuracies when the subspace *mtry* = ⌈log⁡_2_(*M*) + 1⌉ was used. However, the xRF model is better than the wsRF model in increasing the prediction accuracy on the three classification datasets. The RF model requires the larger number of features to achieve the higher accuracy of prediction, as shown in the right of Figures 9(a) and 9(b). When the number of trees in a forests was varied, the xRF model produced the best results on the* Fbis* and* La2s* datasets. In the* La1s* dataset where the xRF model did not obtain the best results, as shown in Figure 9(c) (left), the differences from the best results were minor. From the right of Figures 9(a), 9(b), and 9(c), we can observe that the xRF model does not need many features in the selected subspace to achieve the best prediction performance. These empirical results indicate that, for application on high-dimensional data, when the xRF model uses the small subspace, the achieved results can be satisfactory.

However, the RF model using the simple sampling method for feature selection [1] could achieve good prediction performance only if it is provided with a much larger subspace, as shown in the right part of Figures 9(a) and 9(b). Breiman suggested to use a subspace of size $mtry=\sqrt{M}$ in classification problem. With this size, the computational time for building a random forest is still too high, especially for large high datasets. In general, when the xRF model is used with a feature subspace of the same size as the one suggested by Breiman, it demonstrates higher prediction accuracy and shorter computational time than those reported by Breiman. This achievement is considered to be one of the contributions in our work.

## 6. Conclusions

We have presented a new method for feature subspace selection for building efficient random forest xRF model for classification high-dimensional data. Our main contribution is to make a new approach for unbiased feature sampling, which selects the set of unbiased features for splitting a node when growing trees in the forests. Furthermore, this new unbiased feature selection method also reduces dimensionality using a defined threshold to remove uninformative features (or noise) from the dataset. Experimental results have demonstrated the improvements in increasing of the test accuracy and the AUC measures for classification problems, especially for image and microarray datasets, in comparison with recent proposed random forests models, including RF, GRRF, and wsRF.

For future work, we think it would be desirable to increase the scalability of the proposed random forests algorithm by parallelizing them on the cloud platform to deal with big data, that is, hundreds of millions of samples and features.

## Figures and Tables

**Algorithm 1:**
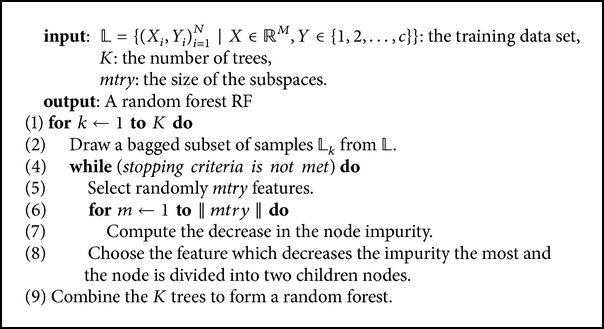
Random forest algorithm.

**Algorithm 2:**
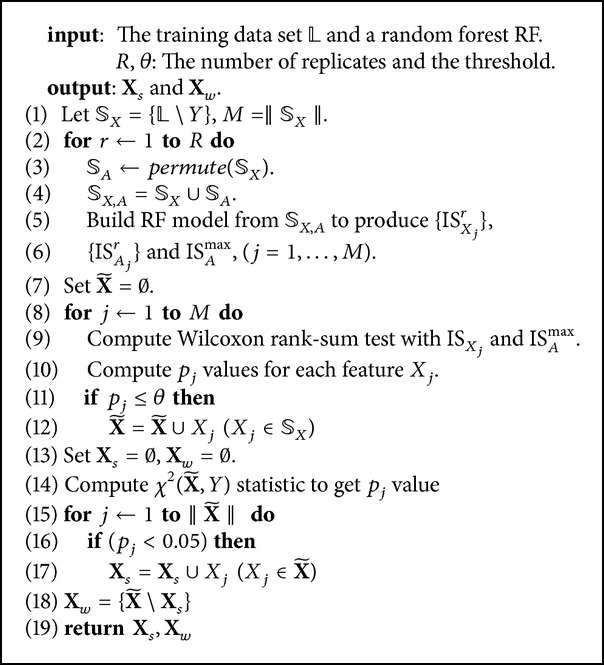
Feature subspace selection.

**Table 1:** Description of the real-world datasets sorted by the number of features and grouped into two groups, microarray data and real-world datasets, accordingly.

Dataset	No. of features	No. of training	No. of tests	No. of classes
Colon	2,000	62	—	2
Srbct	2,308	63	—	4
Leukemia	3,051	38	—	2
Lymphoma	4,026	62	—	3
breast.2.class	4,869	78	—	2
breast.3.class	4,869	96	—	3
nci	5,244	61	—	8
Brain	5,597	42	—	5
Prostate	6,033	102	—	2
adenocarcinoma	9,868	76	—	2
Fbis	2,000	1,711	752	17
La2s	12,432	1,855	845	6
La1s	13,195	1,963	887	6

**Table 2:** The (*c*/*s*2) error bound results of random forest models against the number of codebook size on the Caltech and Horse datasets. The bold value in each row indicates the best result.

Dataset	Model	300	500	1000	3000	5000	7000	10000	12000	15000
Caltech	xRF	** .0312**	** .0271**	.0280	.0287	.0357	.0440	.0650	.0742	** .0789**
	RF	.0369	.0288	.0294	.0327	.0435	.0592	.0908	.1114	.3611
	wsRF	.0413	.0297	** .0268**	** .0221**	** .0265**	** .0333**	** .0461**	** .0456**	.0789

Horse	xRF	** .0266**	** .0262**	** .0246**	** .0277**	** .0259**	** .0298**	** .0275**	** .0288**	** .0382**
	RF	.0331	.0342	.0354	.0374	.0417	.0463	.0519	.0537	.0695
	wsRF	.0429	.0414	.0391	.0295	.0288	.0333	.0295	.0339	.0455

**Table 3:** The prediction test accuracy (mean% ± std-dev%) of the models on the image datasets against the number of trees *K*. The number of feature dimensions in each subdataset is fixed. Numbers in bold are the best results.

Dataset	Model	*K* = 20	*K* = 50	*K* = 80	*K* = 100	*K* = 200
CaltechM3000	xRF	** 95.50 ± .2**	** 96.50 ± .1**	** 96.50 ± .2**	** 97.00 ± .1**	** 97.50 ± .2**
	RF	70.00 ± .7	76.00 ± .9	77.50 ± 1.2	82.50 ± 1.6	81.50 ± .2
	wsRF	91.50 ± .4	91.00 ± .3	93.00 ± .2	94.50 ± .4	92.00 ± .9
	GRRF	93.00 ± .2	96.00 ± .2	94.50 ± .2	95.00 ± .3	94.00 ± .2

HorseM3000	xRF	** 80.59 ± .4**	** 81.76 ± .2**	** 79.71 ± .6**	** 80.29 ± .1**	** 77.65 ± .5**
	RF	50.59 ± 1.0	52.94 ± .8	56.18 ± .4	58.24 ± .5	57.35 ± .9
	wsRF	62.06 ± .4	68.82 ± .3	67.65 ± .3	67.65 ± .5	65.88 ± .7
	GRRF	65.00 ± .9	63.53 ± .3	68.53 ± .3	63.53 ± .9	71.18 ± .4

YaleB.EigenfaceM504	xRF	75.68 ± .1	** 85.65 ± .1**	** 88.08 ± .1**	88.94 ± .0	91.22 ± .0
	RF	71.93 ± .1	79.48 ± .1	80.69 ± .1	81.67 ± .1	82.89 ± .1
	wsRF	** 77.60 ± .1**	85.61 ± .0	88.11 ± .0	89.31 ± .0	90.68 ± .0
	GRRF	74.73 ± .0	84.70 ± .1	87.25 ± .0	** 89.61 ± .0**	** 91.89 ± .0**

YaleB.randomfaceM504	xRF	94.71 ± .0	97.64 ± .0	98.01 ± .0	98.22 ± .0	98.59 ± .0
	RF	88.00 ± .0	92.59 ± .0	94.13 ± .0	94.86 ± .0	96.06 ± .0
	wsRF	95.40 ± .0	97.90 ± .0	98.17 ± .0	98.14 ± .0	98.38 ± .0
	GRRF	** 95.66 ± .0**	** 98.10 ± .0**	** 98.42 ± .0**	** 98.92 ± .0**	** 98.84 ± .0**

ORL.EigenfaceM504	xRF	76.25 ± .6	87.25 ± .3	** 91.75 ± .2**	** 93.25 ± .2**	** 94.75 ± .2**
	RF	71.75 ± .2	78.75 ± .4	82.00 ± .3	82.75 ± .3	85.50 ± .5
	wsRF	** 78.25 ± .4**	** 88.75 ± .3**	90.00 ± .1	91.25 ± .2	92.50 ± .2
	GRRF	73.50 ± .6	85.00 ± .2	90.00 ± .1	90.75 ± .3	94.75 ± .1

ORL.randomfaceM504	xRF	** 87.75 ± .3**	92.50 ± .2	** 95.50 ± .1**	94.25 ± .1	** 96.00 ± .1**
	RF	77.50 ± .3	82.00 ± .7	84.50 ± .2	87.50 ± .2	86.00 ± .2
	wsRF	87.00 ± .5	** 93.75 ± .2**	93.75 ± .0	** 95.00 ± .1**	95.50 ± .1
	GRRF	87.25 ± .1	93.25 ± .1	94.50 ± .1	94.25 ± .1	95.50 ± .1

**Table 4:** AUC results (mean ± std-dev%) of random forest models against the number of trees *K* on the CaltechM3000 and HorseM3000 subdatasets. The bold value in each row indicates the best result.

Dataset	Model	*K* = 20	*K* = 50	*K* = 80	*K* = 100	*K* = 200
CaltechM3000	xRF	** .995 ± .0**	** .999 ± .5**	** 1.00 ± .2**	** 1.00 ± .1**	** 1.00 ± .1**
	RF	.851 ± .7	.817 ± .4	.826 ± 1.2	.865 ± .6	.864 ± 1
	wsRF	.841 ± 1	.845 ± .8	.834 ± .7	.850 ± .8	.870 ± .9
	GRRF	.846 ± .1	.860 ± .2	.862 ± .1	.908 ± .1	.923 ± .1

HorseM3000	xRF	** .849 ± .1**	** .887 ± .0**	** .895 ± .0**	** .898 ± .0**	** .897 ± .0**
	RF	.637 ± .4	.664 ± .7	.692 ± 1.5	.696 ± .3	.733 ± .9
	wsRF	.635 ± .8	.687 ± .4	.679 ± .6	.671 ± .4	.718 ± .9
	GRRF	.786 ± .3	.778 ± .3	.785 ± .8	.699 ± .1	.806 ± .4

**Table 5:** Test accuracy results (%) of random forest models, GRRF(0.1), varSelRF, and LASSO logistic regression, applied to gene datasets. The average results of 100 repetitions were computed; higher values are better. The number of genes in the strong group **X**_*s*_ and the weak group **X**_*w*_ is used in xRF.

Dataset	xRF	RF	wsRF	GRRF	varSelRF	LASSO	**X**_*s*_	**X**_*w*_
colon	** 87.65**	84.35	84.50	86.45	76.80	82.00	245	317
srbct	97.71	95.90	96.76	97.57	96.50	** 99.30**	606	546
Leukemia	89.25	82.58	84.83	87.25	89.30	** 92.40**	502	200
Lymphoma	** 99.30**	97.15	98.10	99.10	97.80	99.10	1404	275
breast.2.class	** 78.84**	62.72	63.40	71.32	61.40	63.40	194	631
breast.3.class	** 65.42**	56.00	57.19	63.55	58.20	60.00	724	533
nci	** 74.15**	58.85	59.40	63.05	58.20	60.40	247	1345
Brain	** 81.93**	70.79	70.79	74.79	76.90	74.10	1270	1219
Prostate	92.56	88.71	90.79	** 92.85**	91.50	91.20	601	323
Adenocarcinoma	** 90.88**	84.04	84.12	85.52	78.80	81.10	108	669

**Table 6:** The accuracy of prediction and error bound *c*/*s*2 of the models using a small subspace *mtry* = [log_2_⁡(*M*) + 1]; better values are bold.

Dataset	*c*/*s*2 Error bound			Test accuracy (%)				**X**_*s*_	**X**_*w*_
	RF	wsRF	xRF	RF	GRRF	wsRF	xRF		
Fbis	.2149	**.1179**	.1209	76.42	76.51	84.14	**84.69**	201	555
La2s	152.6	.0904	**.0780**	66.77	67.99	87.26	**88.61**	353	1136
La1s	40.8	**.0892**	.1499	77.76	80.49	86.03	**87.21**	220	1532
